# Taxicab tipping and sunlight

**DOI:** 10.1371/journal.pone.0179193

**Published:** 2017-06-08

**Authors:** Srikant Devaraj, Pankaj C. Patel

**Affiliations:** 1 Center for Business and Economic Research, Miller College of Business, Ball State University, Muncie, Indiana, United States of America; 2 Villanova School of Business, Villanova University, Villanova, Pennsylvania, United States of America; Mälardalen University, SWEDEN

## Abstract

Does the level of sunlight affect the tipping percentage in taxicab rides in New York City? We examined this question using data on 13.82 million cab rides from January to October in 2009 in New York City combined with data on hourly levels of solar radiation. We found a small but statistically significant positive relationship between sunlight and tipping, with an estimated tipping increase of 0.5 to 0.7 percentage points when transitioning from a dark sky to full sunshine. The findings are robust to two-way clustering of standard errors based on hour-of-the-day and day-of-the-year and controlling for day-of-the-year, month-of-the-year, cab driver fixed effects, weather conditions, and ride characteristics. The NYC cab ride context is suitable for testing the association between sunlight and tipping due to the largely random assignment of riders to drivers, direct exposure to sunlight, and low confounding from variation in service experiences.

## Introduction

Tipping is an area of increasing interest in economics and social sciences [1–3] and represents a significant portion of the United States economy. The US food industry alone generated $46.6 billion in tips in 2011 [4]. The present study proposed both economic and noneconomic rationales for tipping. The economic rationale for tipping is to reward service quality and receive better future service [1, 5]. Tipping also has positive externalities on other customers. However, tips are not legally required and represent an unnecessary cost to customers. Supporting noneconomic rationale for tipping, Lynn and McCall [6] found a near-zero correlation between tip size and service evaluations and variability of less than two percent in tipping amounts. A lower correlation between tip size and service evaluation implies that customers could be primed by noneconomic motives for tipping. Rationales for tipping in the social psychology literature include doing a good thing, reducing guilt, abiding by social norms, and empathy and compassion toward service workers, among others [7]. Whereas an individual’s personality (e.g., individuals with greater empathy are likely to tip higher in different service contexts) and social conditioning (e.g., willingness to follow tipping norms) are generally stable, tipping could also be influenced by more transient factors such as weather-induced moods.

Weather explains significant variance in mood, and among weather-related factors, sunlight has the most immediate and more lasting impact on mood [3, 8]. The seemingly benign influence of weather is well-documented in behavioral economics. Weather has been shown to influence stock market returns [7], prices paid at art auctions in London [9], car purchases [10], returns of products purchased from catalogs [11], worker productivity [3], loan decisions [12], and the assessment of a company’s earnings reports [3], among others. Sunny days induce a positive mood, and a positive mood could potentially increase tipping [5, 13–15]. Some studies have found support for the relationship between sunlight and tipping [16–18], whereas others have found no association [19]. In a quantitative aggregation of two studies on sunshine and tipping, Lynn and McCall [20] found a small- to medium effect size between bill-adjusted tips and sunny weather.

To address the mixed findings on the sunlight—tipping association, the purpose of the present paper is to assess the association between hourly variation in solar radiation and tipping percentage in NYC taxicab rides. The NYC cab ride tipping context is meaningful from both practical and empirical perspectives. From a practical point, NYC taxicabs represent a significant portion of NYC’s economy—13,437 medallions are worth about $800,000 each; cabs complete 485,000 rides per day; 50,000 active drivers work in 9.5 hour shifts on average and transport 600,000 passengers per day. This equates to 236 million passengers per year. Empirically, the taxicab tipping context allows for the robust testing of the sunlight and tipping association for several reasons. First, the cab ride experience is generally standardized (versus other service settings in which type of service, décor, and the status of the service establishment confound with tipping). Second, service evaluations are less likely to vary across cab riders experiencing this standardized cab service, leading to a lower confound between variations in service evaluation and tipping. Third, exposure to sunlight is direct in a cab (compared to enclosed spaces such as restaurants). Finally, sorting between drivers and riders is limited, compared to when higher-quality service workers sort into establishments in which higher tips are likely [13]. These advantages allow for a quasi-natural experiment-type setup, in which drivers and riders are almost randomly matched. The influence of treatment (sunlight) is exogenously varying on an hourly basis and is less confounded by variations in service quality across encounters. Below, we further elaborate on the benefits of the NYC taxicab tipping context in testing the sunlight—tipping association.

Past work on tipping shows that service providers play an important role in altering tipping behavior. Gestures, tone, affect, and actions of restaurant servers have a strong effect on tips [1, 15]. In taxicabs, the effects of such behaviors and actions are limited except possibly for tone and the nature of the occasional conversation between cab drivers and customers. Taxicab services are standardized, and cab drivers do not have significant control over enhancing the service experience. Anecdotal evidence suggests that cab riders focus on baseline hygiene factors and are not particularly motivated to go above and beyond basic service levels [21]. In a service setting that a customer visits often, there is a stronger economic incentive to tip with the expectation of receiving better future services [22]. However, a cab rider is less likely to encounter the same cab driver again; therefore, the expectation of a future meeting would not drive the tipping percentage. Nor are there positive externalities from tipping, as tipping cab drivers does not improve the overall service delivery of other cab drivers. Thus, taxicab rides lower confounding from variation in service experiences. While the friendliness of a cab driver is unobserved and could influence tipping, we control for the fixed effects of cab drivers.

Past studies on weather and tipping behavior have tested the effects of weather on tipping in enclosed spaces such as restaurants [23, 24], where customers are not exposed directly to sunlight. Whether customers in enclosed spaces pay attention to outside weather during service encounters is also affected by customers’ unobservables, such as their attention span. The treatment of sunlight, however, is present throughout the cab ride and does not vary from one cab to another, because NYC cabs are not allowed to tint their windows for both passenger and cab driver safety. The current study is the first large-scale field study to assess the direct effects of hourly variation in sunlight on tipping behavior.

Most important, the key confound biasing tipping estimates could be sorting between customers and service workers. Higher quality servers sort into higher paying restaurants, and clients with a greater willingness to spend more money (and therefore tip) are more likely to visit high-end restaurants [5]. Sorting from demand- and supply side are less plausible for cab rides. According to the 2014 NYC Taxicab Factbook, the average trip distance is 2.6 miles, and the cost of an average cab ride, except for longer distances, does not differ substantially from other modes of transportation (e.g., bus or subway). Almost 90 percent of NYC taxicab pickups are in Manhattan, where most trips are less than a few blocks. Due to shorter distances and the majority of cab rides being concentrated in Manhattan, where the easiest mode of transportation is a cab, selection effects from customers based on the cost of transportation are likely to be lower. Furthermore, riders cannot request cabs based on a driver’s ability or the distance they want to travel or the friendliness of cab drivers.

Limited sorting also exists on the supply side. Except for anecdotal evidence in studies on cab drivers in NYC who are less willing to stop for African-American or other minority riders, cab drivers pick up passengers through street hails or e-hails, when notified by dispatch, or based on their number in the pickup queue at the airport. Cab drivers cannot sort themselves in picking higher-fare customers, because they cannot deny fare based on the distance of travel. Thus, cab drivers do not actively manage the selection of customers. Overall, due to quasi-randomness in rider—driver matching, we do not expect the estimates to be significantly biased to the possibility of two-sided sorting.

Finally, Azar [25] found that tipping behaviors could change based on changing social norms. Such changes in tipping norms are not present in the current sample, as the NYC Taxicab Factbook [26] states that “tipping by taxi passengers has remained rather constant for the last few years, holding at an average tip of 18%” (page 7). The previous NYC Taxicab Factbook published in 2006 assumes average tips to be at 15% during 1990 and 2005 [7]. Based on these two reports, the tipping norm for NYC cab rides has ranged from 15% to 18%, and the data in this study show average tips to be around 19%, indicating limited, if any, change in tipping norms for taxicabs.

Overall, the proposed empirical setup allows for a quasi-natural experiment-type setup to study the influence of hourly levels of sunlight on tipping in individual cab rides. The remainder of the present paper is organized as follows. We start by discussing past work on tipping followed by the influence of sunlight on mood. Thereafter, we provide plausible pathways for a positive association between sunlight and tipping. Next, we elaborate on the cab-ride level sample and present the analyses. Finally, we summarize the results and discuss directions for future research.

## Conceptual framework

### Tipping literature

Tips are voluntary payments customers make to service workers who perform services for them [3]. Numerous factors, including service quality, bill size, party size (e.g., number of customers at a restaurant table), social benefits of service after the encounter (e.g., haircuts), and time of the day (tips tend to be larger in the evening), influence tipping. Some customers leave a flat dollar amount tip, others tip based on a percentage, whereas others leave a minimum tip irrespective of the bill amount [3]. In the United States, the norm of tipping is between 15 and 20 percent, and tipping represents a significant portion of the informal economy [13]. Understanding tipping behavior has been an area of academic interest over the past three decades. Below, we briefly review this literature. For a more detailed review of tipping literature, we refer readers to [15, 27, 28].

Tipping could be motivated by both economic and noneconomic reasons. Economic motivations for tipping have been studied using rational choice theory and equity theory [29]. Tipping is counterintuitive to the rational choice theory, because voluntary payments (tips) after receiving services do not directly influence the utility received from services. However, others have demonstrated that higher utility from tipping is driven by service quality or the expectation of receiving better future service [27]. Equity theory explains tipping based on service quality, higher the ratio of service experiences of customers (outputs [e.g., service quality]) to the inputs service workers provided, higher the tip [27]. Additional economic rationales for tipping include prices of services availed [28], bill size [29], patronage frequency [28], risk-sharing with the customer [30], paying by credit card [14], and myopia in spending induced by alcohol consumption [13], among others.

Noneconomic reasons for giving higher tips include abiding by social norms, increasing social esteem and social status, and enhancing server welfare, among others. Tipping is largely a norm-driven behavior [3]. Variations in cultural norms across countries also influence tipping [31]. Customers leave a tip to adhere to social norms or to reduce guilt [32]. Customers also tip to maintain social status and improve feelings of self-esteem [33]. Tipping, as a tool to display individual status, allows customers to demonstrate their socioeconomic status to themselves, their guests, or to service workers [34]. Although there are differing accounts as to whether tipping started in 16^th^ Century Europe or whether it was prevalent in Roman times [35], scholars agree that tipping originated as a feature of socioeconomic status [36], whereby individuals of economically higher status (i.e., wealthy) gave tips to people of economically lower status. Because service workers are low-wage employees for whom tips are a major income source, leaving higher tips could improve server welfare [27]. Additional noneconomic reasons for tipping include the service worker’s attractiveness [27], race [28], gender [29], and clothing color [37].

Another aspect that influences tipping is the weather [35]. In the tipping literature, on spring days, tips in Chicago-area restaurants were higher [38], and the weather is positively related to tipping [9]. However, in a two-year longitudinal data of 11,766 credit card receipts of a non-chain restaurant in Poughskeepsie, New York, the authors found no relationship between daily sunshine and daily tipping [7]. In their recent meta-analysis, Lynn and McCall [39] found that “consumers left larger bill-adjusted tips under conditions known to elevate mood … when the weather was sunny (r = .20; z = 3.05, *p* < .01; n = 2)” (page 24).

Building on these mixed findings on the sunlight—tipping association, we test this association in the NYC taxicab context with lower confounding from a variety of contextual factors and where two-sided sorting between customers and service providers is negligible. Next, we discuss the nature of the ordinal treatment of sunlight on human mood and behavior, followed by rationales for a positive association between sunlight and tipping.

### Ordinal treatment of sunlight

Sunlight measured at the ground level is a combination of direct sunlight, diffused solar radiation in the sky, and reflected sunlight. Humans are exposed to both ultraviolet and visible solar radiation. Sunlight, with a visible radiation between wavelengths of 380 to 780 nanometers, operates from the eye to the brain through both visual and non-visual paths [40]. Sunlight expressed in lux, a measure of illuminance, ranges from five lux with the sun at the horizon above thick storm clouds to 120,000 lux at noon. The human body does not respond to continuous treatment of sunlight but to an ordinal treatment (e.g., [28, 29, 30]). From Wikipedia of Daylight: 120,000 lux: Brightest sunlight; 110,000 lux: Bright sunlight; 20,000 lux: Shade illuminated by entire clear blue sky, midday; 1,000–2,000 lux: Typical overcast day, midday; <200 lux: Extreme of darkest storm clouds, midday; 400 lux: Sunrise or sunset on a clear day (ambient illumination); 40 lux: Fully overcast, sunset/sunrise; <1 lux: Extreme of darkest storm clouds, sunset/rise. Also, similar values are available from ‘Illuminance—Recommended Light Level’ from The Engineering Toolbox [http://www.engineeringtoolbox.com/light-level-rooms-d_708.html]. Additional information on the ordinal influence of sunlight on humans is available in [27–29].

The visual pathway through the optic nerve affects “visual performance, perceptual judgments, and cognitions” (page 7) [11].

### Impact of weather on mood and behavior

Increasing evidence shows that weather influences affective states, which in turn accumulate to influence decision-making and behavior [41]. The mood and behavior induced by the weather could deviate from rational behaviors. The psychological and biological effects of sunlight on human behavior are documented in behavioral economics (e.g., [7, 26]) and social psychology (e.g., [3]). In behavioral economics, weather is shown to influence a wide range of outcomes, ranging from stock market returns [7] to prices paid at art auctions in London [9] and from car purchases [10] to college admission decisions [39, 40].

Sunlight induces a positive mood as follows. The non-visual effects of sunlight through the retinohypothalamic tract (RHT) are known to influence both the limbic and endocrine systems. Sunlight, through its effects on both the limbic and endocrine systems, influences mood and cognition [28]. The dominant wavelength of sunlight (477 nanometers) through the retinal pathway modulates suprachiasmatic nuclei (SCN), which in turn regulates hormonal systems, blood pressure, and cognitive functioning [30]. SCN affects pineal glands and restricts conversion of serotonin to melatonin. Serotonin, the “feel good” hormone is associated with happiness, contentment, and relaxation, whereas melatonin, a derivative of serotonin, or the “wonder hormone,” affects sleep patterns and acts as an antioxidant. Despite the presence of air conditioning in certain service contexts (including NYC cab rides), sunlight also affects thermal sensation. The so-called thermic alliethesia refers to the physical response to manage body temperature. Exposure to sunlight increases body temperature. The perceptions of higher temperature could modulate body temperature and blood flow, which reduces stress and increases feelings of happiness and relaxation.

### Positive association between sunlight and taxicab tipping

Based on the above discussion, we propose four possible pathways to explain the positive association between sunlight and tipping. Although we do not observe the mechanisms listed below in the present study, to support our proposition, we connect the literature on sunlight and positive mood to the literature on antecedents to tipping.

First, increasing intensity of sunlight affects SCN functioning, which increases serotonin levels and limits the conversion of serotonin to melatonin, thus increasing an individual’s total levels of happiness, contentment, and relaxation [13, 14]. Greater feelings of happiness increase helping behavior [42] and empathy [43]; indeed, both outcomes are associated with tipping [44]. Extant tipping studies have found that customers tip service workers with the desire to help [1, 45], and helping is the predominant motive for tipping among US customers [46]. The positive mood primed by sunlight could actuate hard-wired neural wiring that triggers empathy [47] and generosity [3]. This could, in turn, drive the motive to help cab drivers by tipping them more.

Second, a positive mood is also associated with optimism [48], which in turn could increase tipping. Tipping represents a significant (10–20%) portion of spending, and the need to tip could vary based on situational feelings toward savings, thereby influencing the tendency to tip more (i.e., spend) or less (i.e., save). Lower concerns for saving for the future [49] and higher temporal discounting induced by optimism could reduce the need for saving; such effects may cause individuals to tip more. Based on equity related explanations for tipping, customers could tip more as the perception of service quality (outputs) could be conflated by higher optimism [50, 51].

Third, a positive mood is positively associated with pro-social behaviors [52] and altruism [53]. Continuing from past work on pro-social behavior and tipping [54, 55], positive mood induced by sunlight could increase the tendency to pro-socially help service workers by giving higher tips. Customers exposed to songs with pro-social lyrics [56] or with altruistic quotations on their checks [57] left higher tip amounts. Pro-social behaviors primed through positive mood resulting from sunshine could impel customers to avoid feelings of guilt [58], and motivate them to increase tips by abiding by or exceeding social norms [1].

Fourth, mood maintenance hypothesis [59, 60], according to Handley, Lassiter, Nickell, and Herchenroeder [61], proposes that “individuals…seek out positive activities while in a happy mood in order to maintain or elevate that mood…[a] tendency may become overlearned and, thus, automated” (page 106). When experiencing a positive mood, individuals prefer to avoid activities that ruin that positive mood. To maintain a positive mood induced from sunlight, customers may less stringently evaluate service outputs. Mood maintenance needs, therefore, may also prime customers to avoid losses by reduce feelings of guilt resulting from not fulfilling their sense of duty or meeting the tipping norms.

Overall, we hypothesize that sunlight could influence tipping percentages for cab rides through multiple pathways. Next, we describe the data and the empirical test.

## Methods

### Data

We use ride-level data on licensed yellow cab rides in NYC available from the Taxi and Limousine Commission (TLC) and used in the study conducted by Haggag and Paci [35]. The full dataset of Haggag, K., Paci, G., 2014. Default Tips. *American Economic Journal*: *Applied Economics* 6, 1–19 is available at https://www.aeaweb.org/articles?id=10.1257/app.6.3.1. Complete data is available for rides between January and October of 2009, from 6 am to 4 pm on weekdays and from 6 am to 8 pm on weekends. Each ride was paid using a credit card, was for less than 100 miles, and the drivers’ shifts were longer than 30 minutes but less than 20 hours. A ride was used only if the base amount (comprised of fare, surcharge, toll, and taxes) matched the fare. NYC TLC does not provide data on rides paid for in cash, because there is no standard means to capture cash transactions.

The final sample aggregated based on driver × month × day × ride-level resulted in 13,820,783 observations across 33,478 cab drivers. We matched this with the hourly solar data from the National Solar Radiation Database. We also matched the daily weather variables such as snowfall, rainfall, and average temperature obtained from the National Climatic Data Center.

We converted hourly solar data available in Watts per meter-square (W/m^2^), or the intensity of solar radiation on a perpendicular surface, to lux, the measure of illumination [1 lux = 1 Watt per meter-squared / 0.0079]. The station for measuring solar radiation is located at John F. Kennedy (JFK) Airport. Because most cab rides occur in Manhattan, and due to the proximity of JFK airport to Manhattan, the data covers hourly sunlight variation for most cab rides in NYC.

#### Dependent variable: Tipping

The outcome variable is the ratio of tip amount to the total fare for the ride.

#### Predictor variable: Lux category

Based on the earlier discussion and in [27–29], we use an ordinal measure of hourly sunlight during a cab ride. Hourly lux values of sunlight for each day-of-the-year measured at JFK airport are coded into ordinal categories as follows: 0 (< 1 lux); 1 (between 1 and 40); 2 (between 40 and 200); 3 (between 200 and 400); 4 (between 400 and 1,000); 5 (between 1,000 and 2,000); 6 (between 2,000 and 20,000); 7 (between 20,000 and 110,000); 8 (between 110,000 and 120,000); and 9 (above 120,000).

Category 1 represents sunlight ranging from light under darkest storm clouds (= 1 lux) to light under fully overcast skies or light at sunset and sunrise (= 40 lux). Category 2 represents sunlight ranging from light under fully overcast skies to light at midday with darkest storm clouds (between 40 and 200 lux). Category 3 represents sunlight ranging from light under darkest clouds at midday to light at sunrise and sunset on a clear day (between 200 and 400 lux). Category 4 represents range of sunlight, from light at sunrise or sunset on a clear day to light under overcast skies at midday (between 400 and 1,000 lux). Category 5 represents sunlight under typical overcast skies at midday (1,000 to 2,000 lux). Category 6 represents sunlight ranging from light under overcast skies at midday to shade illuminated by an entirely clear blue sky at midday (between 2,000 and 20,000 lux). Category 7 represents sunlight ranging from shade illuminated by an entirely clear blue sky at midday to bright sunlight (between 20,000 and 110,000 lux). Category 8 represents brightest sunlight (between 110,000 and 120,000 lux), and Category 9 represents any lux values above 120,000 lux (brightest sunlight). Overall, the predictor variable is an ordinal measure, varying by the hour on a given day-of-the-year.

#### Variation in sunlight treatment for each hour

**Fig 1:**
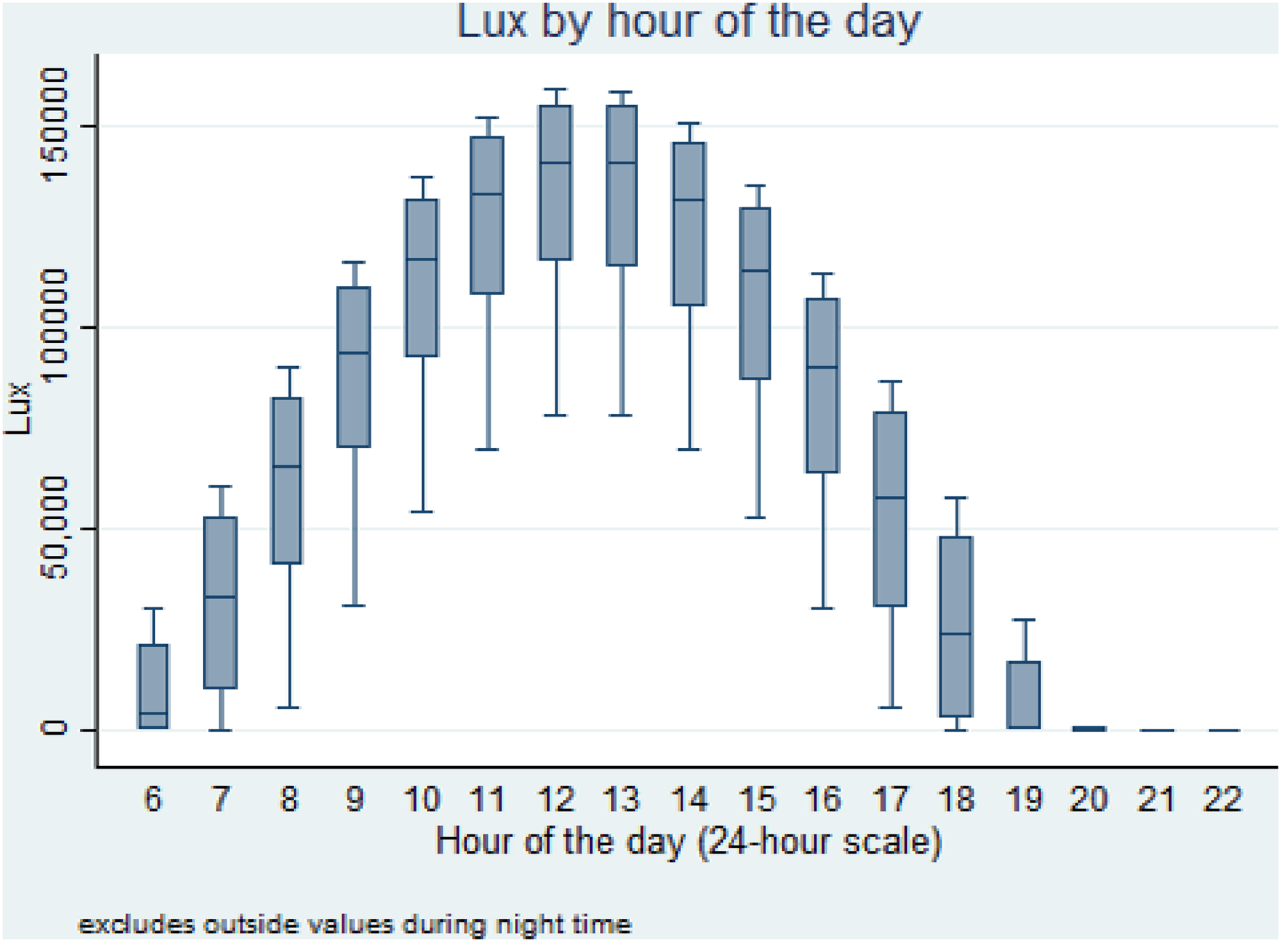
Hourly variation in lux.

To assess whether the ordinal treatment of sunlight levels vary for each hour of the day, we plot the box-whiskers plot of lux during each hour (Fig 1). The variation in lux is downward biased because as the day progresses, the mean sunlight increases. However, varying sunlight due to changing cloud cover shifts the lower extreme of a whisker of box-plot downward. Significant variation in lux values around mean sunlight during each hour of the day (Fig 1), suggests that in the sample, sunlight varies during each hour of the day to plausibly influence tipping. Next, to test our main proposition, we explore the positive association between sunlight and tip fraction.

### Association between sunlight and tip fraction

**Fig 2:**
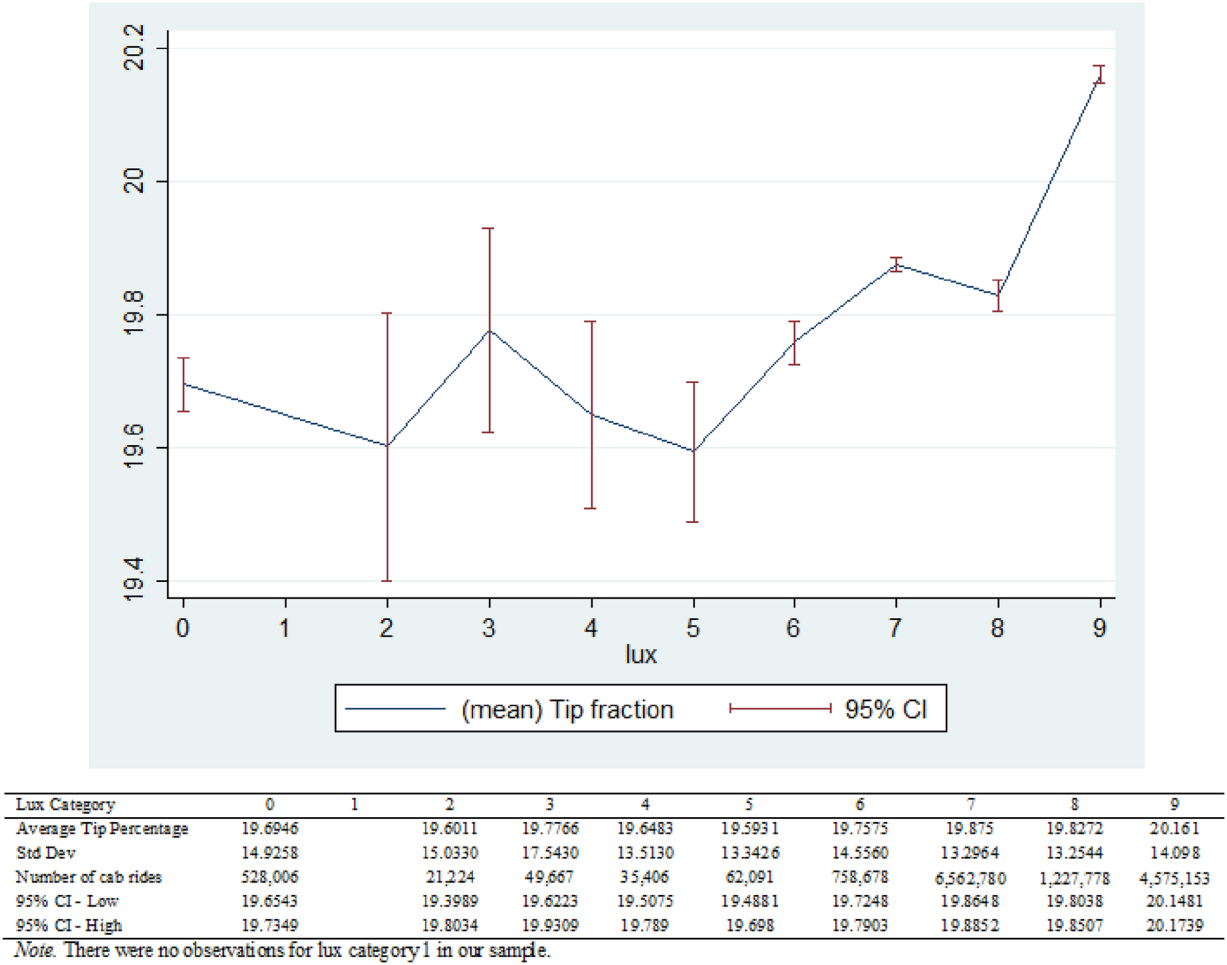
Association between tip fraction and sunlight.

In Fig 2, we plot tip fractions across lux categories. At low lux levels (from dark to overcast skies; lux categories 1 to 5), mean tipping levels are somewhat constant but have large confidence intervals. Thereafter, tipping levels rise with increasing brightness and with tighter confidence intervals. Increasing mean tip fraction in transitioning from overcast to sunny skies and tighter confidence intervals suggest a relative increase of average tips from overcast skies to bright sunlight.

Although Fig 2 implies a non-linear relationship, for simplicity, we will nonetheless model the relationship between lux levels and tipping as linear.

However, the interpretation in Fig 2 could be confounded by a wide range of factors. We explore pairwise correlations of plausible confounds with sunlight and tipping fraction (S1 Table).

A variety of weather-related factors could influence tipping. It is widely documented that the demand for cab rides increases significantly on rainy days or during bad weather [37]. Daily *snowfall* in millimeters (mm) and *rainfall* in tenths of a millimeter (mm) could increase demand of cab rides significantly, increase the propensity to tip, and affect driving conditions. In S1 Table, snow and rainfall are positively associated with tipping fraction. Snow and rainfall are negatively correlated with shorter rides (negative correlation with distance) and extreme temperatures are positively correlated with longer rides.

In addition to the weather variables correlated with tipping, *ride distance* in miles and *ride duration* in minutes could also be correlated with tipping. Longer duration and distance could add to rider frustration and therefore could be negatively correlated with tipping fraction. In S1 Table, these two variables are negatively correlated with tip fraction.

*Passenger count*, defined as the number of passengers in a ride, could prime the need for social esteem (tipping more). For shared rides, customers could perceive more money saved as fare could be shared among customers. In S1 Table, tip fraction is positively correlated with a higher passenger count.

During the period represented in the sample, two tipping systems were used in NYC cabs [35]. Although both systems provide credit card processing systems, during the study period, the tips were computed differently. The vendor system offers default options of $2/$3/$4 for fares below $15 and 20%, 25%, and 30% for fares above $15. A competitor system offered default tipping options of 15%, 20%, and 25% on the sum of the fare, surcharge, taxes, and tolls. *Default tip* option (10%, 15%, and 20% tip) used at the end of the ride is positively correlated with a higher tip fraction (S1 Table).

Finally, demand for rides, willingness to tip a higher percentage, and the ability to drive could be affected by rush hour, day of the week, day-of-the-year (e.g., generosity during the Christmas holidays or frustration during traffic due to the New York Marathon), and month-of-the-year. These factors are correlated with tip fraction at varying levels in S1 Table. Finally, although driver effects cannot be meaningfully captured in a correlation using driver identification, driver’s conversational abilities, and driving skills, among other factors could be associated with ride experience and tip.

### Empirical specification

Based on the above discussion on S1 Table, we propose the following specification, which controls for a variety of confounds:

$$T_{ijhdm}=\alpha_{ijhdm}+\beta_{h}Lux_{hd}+\beta_{d}D_{d}+\beta_{j}C_{ij}+\beta_{r}R_{i}+Driver_{j}+Day_{d}+Month_{m}+\varepsilon_{ijhdm}$$

Where *T*_*ijhdm*_ is the percentage tip received relative to total fare during ride *i*, by driver *j*, in hour *h*, on day *d*, in month *m* during the year. *Lux*_*hd*_ is the lux category during the hour on day *d* of the year. *D*_*d*_ is the vector of day-level predictors—snowfall, rainfall, average temperature during the day, and the square of average temperature during the day. Cab-level predictors (*C*_*j*_) include whether the cab used the vendor’s or the competitor’s passenger information system. Ride-level predictors (*R*_*i*_) include ride duration, ride distance, number of passengers, whether the default menu tip was used, ride during rush hour, and ride during a weekday. We also include driver (driver ID), day-of-the-year, and month-of-the-year fixed effects.

*ε*_*ijhdm*_ is the error term of the regression model. The standard errors are clustered by the combined day-of-the-year and hour-of-the-day. This allows for a within-cluster error arbitrary correlation among our observations.

Variable definitions and descriptive statistics of the sample are presented S2 Table. Average tip fraction was 19.95% with an inter-quartile range of 14.90% to 23.29%. The average distance of a ride was 2.57 miles, with an inter-quartile range from 1.18 miles to 3.10 miles. The average ride duration was 12.77 minutes, and the median ride duration was 11 minutes. An average ride had 1.61 passengers, with 73.9% of rides having a single passenger. Although NYC taxicabs have a default tipping option of 10%, 15%, and 20% or a suggested dollar amount, passengers used the default tipping option only 52.38% of the time. Note, too, that the sample includes only rides paid by credit card). Passengers made a tipping decision that was not based on set norms of tipping 10%, 15%, or 20% 47.62% of the time. Most the rides occurred during weekdays (71.74%), and the average lux was between 20,000 and 120,000 lux across different hours of the day.

## Results

Table 1 presents the estimation results of various covariates on tipping percentage. Model 1 shows our baseline results without any fixed effects. Model 2 presents baseline results with driver fixed effects. Model 3 presents results with driver and day-of-the-year fixed effects. Model 4, our preferred specification, includes driver, day-of-the-year, and month fixed effects.

**Table 1:** Estimation results for impact of lux on tipping.

	(1)	(2)	(3)	(4)
VARIABLE	Tip percentage	Tip percentage	Tip percentage	Tip percentage
Lux category (0 to 9)	0.0429***	0.0580***	0.0702***	0.0702***
	(0.0051)	(0.0050)	(0.0051)	(0.0051)
Snowfall	0.0029***	0.0027***	–0.0174	–0.0188
	(0.0008)	(0.0008)	(0.0193)	(0.0192)
Rainfall	0.0006***	0.0006***	0.0070	0.0076
	(0.0001)	(0.0001)	(0.0058)	(0.0058)
Average daily temperature	0.0008	0.0013	–0.5325**	–0.5906**
	(0.0024)	(0.0023)	(0.2501)	(0.2550)
Average daily temperature-squared	–0.0000	–0.0000	0.0087**	0.0094**
	(0.0000)	(0.0000)	(0.0037)	(0.0038)
Ride duration	–0.3329***	–0.3427***	–0.3456***	–0.3456***
	(0.0017)	(0.0016)	(0.0016)	(0.0016)
Ride distance	–0.1321***	–0.1310***	–0.1224***	–0.1224***
	(0.0044)	(0.0043)	(0.0043)	(0.0043)
Passenger count	0.0069**	0.0354***	0.0376***	0.0376***
	(0.0032)	(0.0078)	(0.0078)	(0.0078)
Vendor	3.6663***	3.7046***	3.7006***	3.7006***
	(0.0109)	(0.0255)	(0.0255)	(0.0255)
Default tip option used	6.6614***	6.6567***	6.6566***	6.6566***
	(0.0112)	(0.0111)	(0.0111)	(0.0111)
Ride during rush hour	–0.5445***	–0.5009***	–0.4887***	–0.4887***
	(0.0185)	(0.0184)	(0.0171)	(0.0171)
Weekday	0.5910***	0.6096***	1.4416	1.4758*
	(0.0161)	(0.0156)	(0.8796)	(0.8839)
Driver fixed effects	No	Yes	Yes	Yes
Day-of-the-year fixed effects	No	No	Yes	Yes
Month fixed effects	No	No	No	Yes
R-squared	0.107	0.115	0.115	0.115

*Notes*. Standard errors clustered by day-of-the-year and hour-of-the-day are in parentheses.

* *p* < 0.10,

** *p* < 0.05,

*** *p* < 0.01.

Because small *p* values can be an artifact of a large sample size, we took four random samples of 5% of the sample to further reduce the possibility of Type 1 error. The results showed comparable estimates in direction and magnitude. To ensure that the outcome measure was not misspecified the two alternate measures of solar radiation—a continuous measure of hourly lux and hourly solar radiation on a perpendicular surface (Watts per meter-squared) at JFK airport showed similar inferences.

The estimated increase in tipping due to a single step increase in lux category is 0.07 percentage points (95% confidence interval from 0.06 to 0.08). Considering that there are nine ordinal sunlight categories between darkness and full sun, this corresponds to an estimated 0.5 to 0.7 percentage points increase in tipping when going from darkness to full sun. In terms of increase in tipping percentage relative to the tipping norm of 20%, this amounts to a relative increase of 2.5% to 3.5% (0.5% divided by 20% and 0.7% divided by 20%).

Based on the 2014 NYC Taxicab Factbook, an average ride is 2.56 miles long. With 485,000 trips per day, using a conservative fare structure (an initial fare of $2.50, $0.40 per 1/5^th^ mile, assuming no idle time, and no surcharge), the fare per ride without tax would be $5.70 ($2.50 for the first mile + [$0.40 for each 1/5^th^ mile for the remaining 1.6 miles of the 2.6-mile ride]). This indicates an increase in tips by $17,466.10 (95% confidence interval: $13,822.50 to $19,351.50) per day for the transition from 0 to 9, or on the order of several million dollars per year.

## Discussion

Tipping remains an area of research interest in both economics and social psychology. Studies have focused on variations in tipping at the individual, establishment, and national levels [45]. Complementing the rational economic theory that does not fully explain tipping, researchers have proposed a variety of explanations, including improving status, gaining approval, striving for equality in economic transactions, helping others, and improving moods and behavior [23].

We show that the tipping percentage increases by 0.63% when transitioning from overcast conditions to fully sunny skies. This result is based on a tipping context where confounders from variations in service-related factors are limited, exposure to sunlight is direct, and sorting between riders and cab drivers is random. In the current context, while the causal chain of the customer’s physio-psychological response to sunlight is unobservable, as is a service encounter, we controlled for month-of-the-year, day-of-the-year, driver fixed effects, and additional ride-, cab-, and day-level characteristics. The standard errors were also clustered using both hour-of-the-day and day-of-the-year. The present study is one of the very few field studies that draw on a large sample from an unobtrusive context to study the association between sunlight and tipping.

Our findings provide an important extension to prior studies on the weather—tipping association. In one of the earliest studies, Cunningham [3] collated data on daily sunlight levels during 13 spring days to assess the association between tipping across 130 customers (10 customers per day). Although Cunningham [3] found a positive association, the inferences were based on a small sample with limited control for confounders. Other studies have relied on either actual or fake weather reported by the service workers [14] or the effects of future weather expectations on current restaurant tipping [13]. Recently, using two-year data from a single restaurant, Flynn and Greenberg [15] found no association between sunlight and tipping. Despite extensions from Cunningham [3], Rind [14], and Rind and Strohmetz [13], Flynn and Greenberg [15] studied tipping levels at the daily level. Compared to these four studies, the present study’s sample is not only the largest ever to study the sunlight—tipping association, but also measures tipping for each transaction, uses a reliable hourly measure of solar radiation, controls for a variety of confounders, and is less influenced by sorting among customers and employees.

Related to practical implications, although tipping could be influenced by interventions such as normative messages, persuasion, or influences to align individual tipping behaviors with service levels, unobtrusive factors such as weather could also impact tipping behavior. Related to the direct effects of weather, sunny days could subconsciously prime a positive mood that increases the need to tip. Messages identifying, guiding, or norming individual behaviors could be better received on sunny days, and customer interactions could be tailored to prime such behaviors when they might be in a more positive mood on sunny days.

The present study’s finding must be interpreted considering its limitations, which also provide directions for future research. First, whereas the current findings from a quasi-natural experimental setting are robust to alternate specifications and a variety of controls, the nature of service interactions is not captured in the data. As such, future studies could focus on lab experiments to further understand the behavioral drivers in the sunlight—tipping association. Such lab studies could also help shed additional light on the continuous treatment effect of sunlight. Furthermore, field studies could be conducted to understand specific moods and motivations of customers. As such, the micro-dynamics of sunlight, associated behavioral changes, and the resulting effect on tipping could be studied in lab settings.

Second, the findings cannot be generalized beyond NYC cab rides during the period of observation, because the weather patterns, urbanization levels, and other factors (e.g., the introduction of Uber) could influence the nature of cab services and competition. The sample is from 2009, when ride-sharing services such as Uber were not present in NYC. Therefore, temporal generalization may not be made after 2009 and is left for future work. Cab data available for Chicago [Source: http://digital.cityofchicago.org/index.php/chicago-taxi-data-released/] and Singapore [62], as well as recently released Uber data, could also add to the generalizability of the finding. Continuing from the lab and field studies described above, cultural and situational characteristics varying across regional and global contexts could further explain tipping variations.

Third, the findings cannot be generalized to other tipping contexts such as restaurants. The service setting for taxicabs is rather unique, where richer service interactions do not occur between customers and service providers. Despite this limitation, as aforementioned, the cab context has several unique advantages that facilitate robust inference for the sunlight—tipping association. Quasi-random matching between riders and drivers, the limited need to tip more generously to get better service in the future, the limited need to be nice based on patronage, limited variation in service provision across service providers are some of the strengths of the studied context. We call on future research to combine this experimental research on tipping with field settings to study tipping behavior in alternate service contexts.

An additional limitation could also be that the findings are a result of noise; that is, noise around the standard tipping norms could be driving the results. Although we control for the default tipping option, it is possible that the identified relationship could be an artifact of noise in a steady-state system consisting of tipping variations across millions of cab rides. However, because a variety of factors such as month- and day-of-the-year fixed effects, including driver fixed effects, are controlled for, the findings may not be the result of model misspecification; however, we do not completely rule this out.

Future research could also draw on machine learning methodologies to understand complex associations among the included variables in the present study. Although the purpose of the present study was to address a simple question on the sunlight—tipping association, the supervised learning method used here (regression) could be augmented using unsupervised learning methods to create latent groups of customers. This would develop a richer understanding of the possible subclasses in the current data. Machine learning approaches could also be used to understand the cab drivers’ decision-making based on past tips and ride performance—whether poorer ride performance or lower aggregate tips affect pickup locations or driving performance. For such an analysis, random forest techniques would be particularly helpful in learning behaviors that could increase fares and tips. Such decision tree methodologies could include pickup locations, day and hour, and ride distance and duration characteristics. With the availability of richer data on driver and passengers, methods such as Bayesian Additive Regression would also be particularly useful in identifying key treatment effects from a variety of cab driver and passenger attributes.

## Supporting information

S1 Table Pairwise correlations. (PDF)

S2 Table Descriptive statistics (*n* = 13,820,783 observations across 33,478 drivers). (PDF)
