# Supplementary material for: Taxicab tipping and sunlight
Source: PLoS One. 2017 Jun 8;12(6):e0179193. doi: 10.1371/journal.pone.0179193 (PMC5464625; doi:10.1371/journal.pone.0179193)
Supplement: S1 Table — (PDF) [file pone.0179193.s001.pdf]

S1 Table. Pairwise correlations

	1	2	3	4	5	6	7	8	9	10	11	12	13
1. Tip percentage	1												
2. Lux category	0.0087	1											
3. Snowfall	0.0077	-0.0844	1										
4. Rainfall	0.0008	0.0527	0.0851	1									
5. Average daily temperature	-0.0096	0.3562	-0.2306	0.0951	1								
6. Average daily temperature-squared	-0.0082	0.3453	-0.2031	0.0785	0.9866	1							
7. Ride distance	-0.1453	-0.0428	-0.0049	-0.0138	0.009	0.0094	1						
8. Ride duration	-0.1837	0.0513	-0.0195	0.0207	0.0389	0.0323	0.7128	1					
9. Passenger count	0.0384	-0.0015	0.0014	-0.0016	**-0.0005**	***0.0001***	0.0117	0.0123	1				
10. Vendor	0.1163	0.0059	0.0021	-0.0013	0.0042	0.0043	0.0138	0.0112	0.3252	1			
11. Default tip option used	0.2138	0.0219	0.0027	**-0.0007**	-0.0074	-0.0063	0.0659	0.1016	-0.0111	-0.061	1		
12. Ride during rush hour	-0.0209	-0.1933	-0.0024	0.0058	-0.0088	-0.0093	0.0022	**-0.0006**	-0.0358	-0.0024	-0.0387	1	
13. Weekday	0.0045	0.1714	0.0076	0.0171	-0.018	-0.019	-0.0611	0.0464	-0.0474	-0.0037	-0.0043	0.3843	1
14. Month	-0.0086	0.1406	-0.1705	0.0963	0.6621	0.6268	-0.005	0.043	-0.0044	0.0025	-0.0084	0.0038	0.0202

Notes.

Bold and italicized estimates is not significant at 10% level.

All remaining estimates are significant at 1% level, except bold typeface estimates are significant at 5% level or 10% level.
