# Supplementary material for: Taxicab tipping and sunlight
Source: PLoS One. 2017 Jun 8;12(6):e0179193. doi: 10.1371/journal.pone.0179193 (PMC5464625; doi:10.1371/journal.pone.0179193)
Supplement: S2 Table — (PDF) [file pone.0179193.s002.pdf]

S2 Table. Descriptive Statistics (*n* = 13,820,783 observations across 33,478 drivers)

**Variable**	**Description**	**Mean**	**Std Dev**	**Min**	**Max**
**Outcome variable**					
Tip percentage	Ratio of tip received to total fare	19.9495	13.7200	0	3900
**Independent Variables**					
Day-level predictors					
Lux Category	Lux category [0 to 9]; lux=0 lux value <1<br>lux=1 between 1 & 40<br>lux=2 between 40 & 200<br>lux=3 200 &400<br>lux=4 400 & 1000<br>lux=5 1000 & 2000<br>lux=6 2000 & 20000<br>lux=7 20000 & 110000<br>lux=8 110000 & 120000<br>lux=9 >120000	7.3899	1.8234	0	9
Snowfall	Snowfall in millimeter	1.4108	11.3087	0	165
Rainfall	Precipitation in tenths of millimeter	40.8984	91.5003	0	584
Average daily temperature	Average daily temperature in degrees Fahrenheit	57.0860	15.9027	12.47	83.48
Average daily temperature—squared	Square of average temperature	3511.7020	1674.7030	155.5009	6968.9110
Cab-level predictor					
Vendor	Reference category<br>Vendor = 1; Competitor = 0	0.5266	0.4993	0	1
Ride-level predictors					
Ride duration (minutes)	Ride duration in minutes	12.7692	8.0291	0.0167	180
Ride distance (mile)	Ride distance in miles	2.5655	2.3586	0.01	49.6
Passenger count	Number of passengers in a ride	1.6065	1.2480	0	7
Default tip option used	Whether the passenger used default tip option.	0.5238	0.4994	0	1
Ride during rush hour	Ride occurred during weekdays between 7 am and10 am and between 4 pm and 7 pm	0.2726	0.4453	0	1
Weekday	Ride occurred during the weekday	0.7174	0.4503	0	1

Note. The included fixed effects are driver (*driver id*), month of the year, and day of the year. Only a couple of rides had 7 passengers.
